# Integrated analysis of lncRNAs and mRNAs reveals key *trans*-target genes associated with ETEC-F4ac adhesion phenotype in porcine small intestine epithelial cells

**DOI:** 10.1186/s12864-020-07192-8

**Published:** 2020-11-10

**Authors:** Serafino M. A. Augustino, Qinglei Xu, Xueqin Liu, Siyuan Mi, Liangyu Shi, Yibing Liu, Hui Wen, Di Wang, Lei Liu, Qin Zhang, Ying Yu

**Affiliations:** 1 Key Laboratory of Animal Genetics, Breeding and Reproduction, Ministry of Agriculture & National Engineering Laboratory for Animal Breeding, College of Animal Science and Technology, China Agricultural University, Beijing, 100193 P. R. China; 2 School of Natural Resources and Environmental Studies, University of Juba, Juba, South Sudan P.O. Box 82,; 3 College of Animal Science and Technology, Nanjing Agricultural University, Nanjing, 210095 China; 4 Chinese Academy of Agricultural Sciences Institute of Animal Science, Beijing, 100193 China; 5 Research Centre for Animal Genomic, Agricultural Genomic Institute at Shenzhen, Chinese Academy of Agricultural Sciences, Shenzhen, 518124 China

**Keywords:** Small intestine epithelial cells, lncRNA *cis*-acting, lncRNA *trans*-acting, Adhesion phenotype, Piglets’ diarrhea susceptibility, ETEC-F4ac

## Abstract

**Background:**

Long non-coding RNAs (lncRNAs) play crucial roles in gene regulation at the transcriptional and post-transcriptional levels. LncRNAs are belonging to a large class of transcripts with ≥200 nt in length which do not code for proteins, have been widely investigated in various physiological and pathological contexts by high-throughput sequencing techniques and bioinformatics analysis. However, little is known about the regulatory mechanisms by which lncRNAs regulate genes that are associated with Enterotoxigenic *Escherichia coli* F4 fimbriae (ETEC-F4ac) adhesion phenotype in small intestine epithelial cells of Large White piglets. To address this, we used RNA sequencing to profile lncRNAs and mRNAs of small intestine epithelial cells in Large White piglets differing in their ETEC-F4 adhesion phenotypes and *ITGB5* genotypes. Eight male piglets were used in this study and were divided into two groups on the basis of their adhesion phenotype and *ITGB5* genotypes, a candidate gene for F4ac receptor. Non-adhesive group (*n* = 4) with CC genotype and adhesive group (*n* = 4) with TT genotype.

**Results:**

In total, 78 differentially expressed lncRNAs (DE-lncRNA) and 223 differentially expressed mRNAs (log2 |FC| > 1, *P* < 0.05) were identified in the comparison of non-adhesive vs. adhesive small intestine epithelial cells. Furthermore, *cis*- and *trans*-regulatory target genes of DE-lncRNAs were identified, then interaction networks of lncRNAs and their *cis-* and *trans*-target differentially expressed genes (DEGs) were constructed separately. A total of 194 *cis*-targets were involved in the lncRNAs-*cis* genes interaction network and 61 *trans*-targets, were involved in lncRNA-*trans* gene interaction network that we constructed. We determined that *cis*-target genes were involved in alcoholism, systemic lupus erythematosus, viral carcinogenesis and malaria. Whereas *trans*-target DEGs were engaged in three important pathways related to the ETEC-F4 adhesion phenotype namely cGMP-PKG signaling pathway, focal adhesion, and adherens junction. The *trans*-target DEGs which directly involved in these pathways are *KCNMB1* in cGMP-PKG signaling pathway, *GRB2* in focal adhesion pathway and *ACTN4* in focal adhesion and adherens junction pathways.

**Conclusion:**

The findings of the current study provides an insight into biological functions and epigenetic regulatory mechanism of lncRNAs on porcine small intestine epithelial cells adhesion to ETEC-F4-ac and piglets’ diarrhea susceptibility/resistance.

**Supplementary Information:**

The online version contains supplementary material available at 10.1186/s12864-020-07192-8.

## Background

Diarrhea is considered one of major problems in piggery industries worldwide particularly during neonatal and postnatal periods and is difficult to be thwarted or eliminated. Many pathogens are implicated but, Enterotoxigenic *Escherichia coli* ETEC-F4ac strain is considered the most important enteric pathogens with the highest prevalence rate. It accounts for 56.2 and 24.7% of the cases of diarrhea and death of piglets respectively [1] and inflicting huge economic burdens on swine farms [2].

Long non-coding RNAs (lncRNAs) play vital roles in the transcriptional and post-transcriptional regulation. Data available from high-throughput sequencing studies have revealed that only a very small percentage (1–2%) of the mammalian genome encode proteins, but tens of thousands of intergenic sites are transcribed to non-coding RNA [3]. This transcription plays a critical role in the regulation of gene expression during several biological processes [4].

LncRNAs belong to a large class of isoforms/transcripts with ≥200 nt in length which do not code for proteins [5, 6], have been widely investigated in various physiological and pathological contexts using high-throughput RNA sequencing (RNA-seq) techniques and bioinformatic analyses. Some lncRNAs are expressed in a highly tissue-specific and cell-type manner, strongly suggesting that lncRNAs may play a cell-specific role [7]. Those lncRNAs, which act in *trans*, exert their function at a different site to which they were transcribed. In contrast, *cis*-acting lncRNAs function by interacting with genes neighboring their site of transcription and can help localize epigenetic modifiers to these locations [8]. Long non-coding RNAs (lncRNAs) have been implicated in the regulation of host inflammatory response against infections caused by enteropathogenic bacteria [9]. For instance, *Salmonella* infection has been demonstrated to be responsible for changes in the expression of certain sensitive lncRNAs in infection in HeLa cells [4].

The overexpression of lncRNA TCONS00183659 in resistant piglets has been reported to regulate the expressions of inflammatory factors IFIT2, MX1 and MX2 to improve the resistance against ETEC-F18 infection in weaning piglet [4]. Similarly, in the ETEC-F18-infected mice diarrhea model, the overexpression of lncRNA ENSMUST00000122226 has been reported to cause improvement in the expression of adhesion molecule CD28 on T cell surface facilitating the secretion of different inflammatory cytokines, including IFN and IL, and thus finally leading to intestinal inflammatory response and diarrhea disease [4] These studies have collectively highlighted the changes and roles of lncRNAs in regulating host inflammatory responses during bacterial infections,

However, the epigenetic regulatory mechanisms by which lncRNAs regulate genes associated with the ETEC-F4ac adhesion phenotype, as a result, the F4ac binds to small intestine epithelial cells and release toxins that cause diarrhea in Large White piglets have not yet been defined.

In order to address this issue, and to evaluate functions of lncRNAs and mRNAs, we performed a comprehensive analysis of the expression profiles of lncRNAs and mRNAs in small intestine epithelial cells of piglets differing in their ETEC-F4 adhesion phenotype and *ITGB5* (a candidate gene of F4ac receptor which plays a key role in *Escherichia coli* F4ac-induced diarrhea in swine) genotype using RNA sequencing. Raw RNA-Seq datasets used in this study were used in our previous published paper [10]. The results could provide support for exploring molecular mechanisms of lncRNAs and mRNAs underlying the ETEC-F4 adhesion phenotype in small intestine epithelial cells of Large White piglets. The aim of this study is therefore to identify lncRNA landscape and integrate them with mRNA to elucidate their regulatory functions in small intestine epithelial cells of Large White piglets differing in their adhesion phenotype to ETEC-F4ac and genotype at *ITGB5* gene. The study detected two lncRNAs *trans*-regulating three genes (*KCNMB1, GRB2*, *ATCN4)* associated with adhesion phenotype in Large White piglets due to their involvement in the cGMP-PKG signaling pathway, focal adhesion and adherent junction respectively.

## Results

### Overview of RNA-sequencing

The Illumina Hiseq Xten platform was used to perform RNA-seq for the eight cDNA libraries, 150 bp paired-end reads were generated. More than 115 million raw reads were generated from each library. After filtering the low-quality reads, reads containing more than 5% Ns, adapter polluted reads, rRNA, and reads with Qphred < 30, the remained clean reads still comprised more than 98% of the raw data. The alignment of the clean reads to the *Sus scrofa* reference genome yielded an average alignment rate of 96.59% with Hisat2–2.1.0. (Additional file 2: Table S1).

### Identification of lncRNAs transcripts and mRNA in porcine small intestine epithelial cells

To investigate the lncRNAs and mRNAs involved in ETEC-F4ac diarrhea susceptibility and development, RNA-seq was performed to identify differentially expressed lncRNAs (DE-lncRNAs) and DEGs in the comparison of ETEC-F4ac non-adhesive vs. adhesive porcine small intestine epithelial cells. To identify lncRNAs (transcripts) and mRNA, Cuffdiff function, the differential expression analysis tool in Cufflinks-2.2.1 software was used. A total of 89,032 transcripts were identified by cuffdiff function in Cufflinks-2.2.1 software. To identify lncRNAs, we used transcripts with length ≥ 200 nt and transcripts with ≥2 exons as criteria, this yielded 87,725 lncRNAs. Subsequently, these lncRNAs (87725) were compared to those reported on the database, 35,026 lncRNAs were found to have NONCODE Transcript ID, whereas 52,699 transcripts had no transcript ID (NA) suggesting they are novel lncRNAs.

**Fig. 1:**
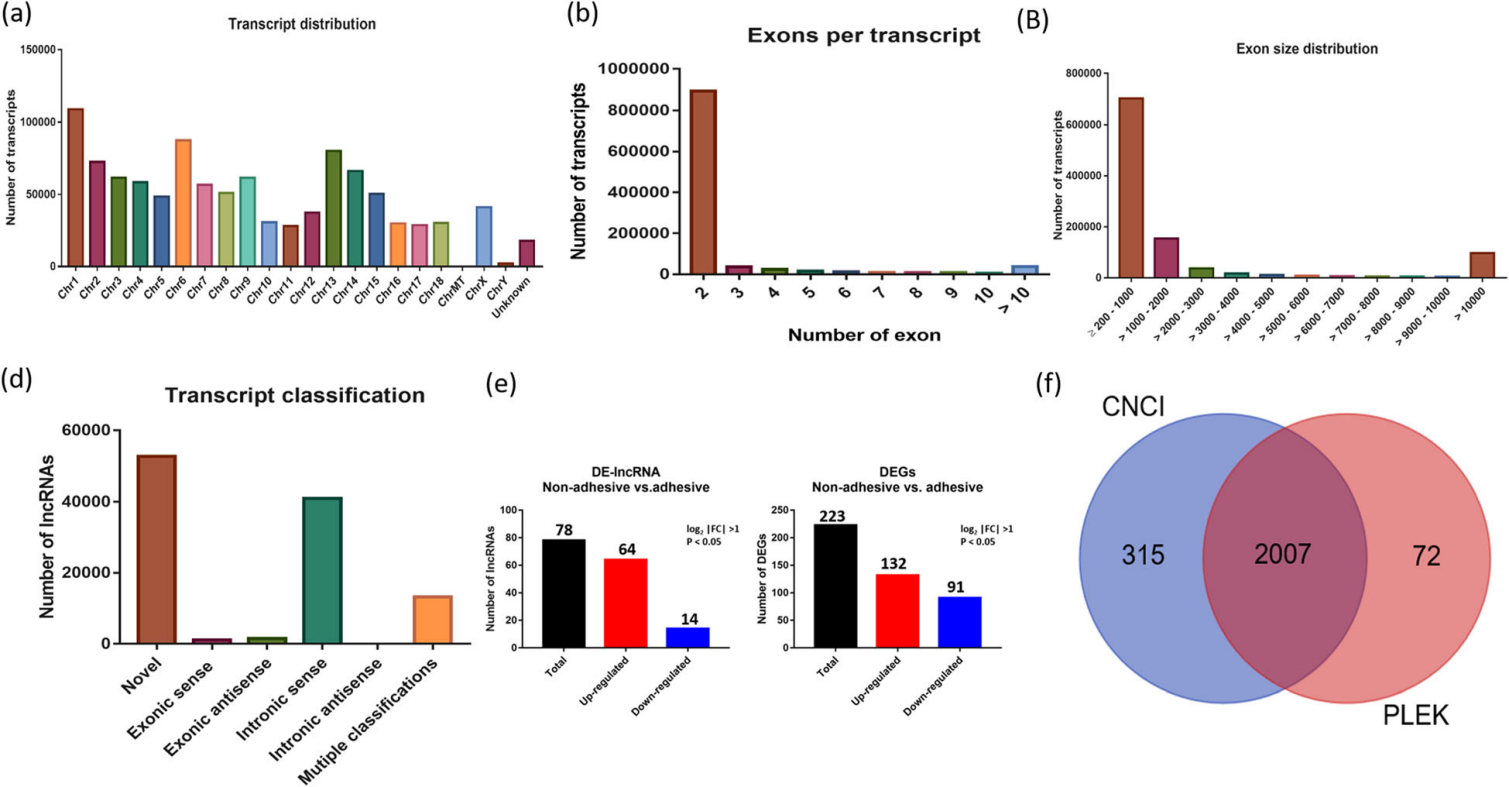
The features of lncRNAs identified in porcine small intestine epithelial cells. **a** Distribution of identified lncRNA transcripts across chromosomes in small intestine epithelial cells of Large White piglets. **b** Exon length distribution of the identified transcripts in small intestine epithelial cells of Large White piglets. **c** Exon numbers per a transcript of lncRNAs in small intestine epithelial cells. **d** Transcript class code distribution. **e** Differentially expressed lncRNA and mRNA in the comparison of non-adhesive Vs. adhesive small intestine epithelial cells. **f** Venn diagram of lncRNAs with coding potential

These transcripts were distributed across all pig chromosomes 1–18, MT, X and Y with the highest numbers 8115 (9.25%) and 7908 (9.01%) seen in Chr6 and Chr1 respectively (Fig. 1a).

The length of the lncRNA transcripts ranged from 200 to 10,391 nt, with 13,228 (15.08%), 1410 (1.60%) and 67,261 (76.67%) having a length of ≥200–1000, > 1000–2000 and > 10,000 nt, respectively (Fig. 1b). The majority of the transcripts (85.11%) have two exons, whereas 3.51% have more than 10 exons (Fig. 1c). A total of 86,055 transcripts were having class code assigned, of which 40,999 (47.64%) were intronic sense and the least 21 (0.0002%) were intronic antisense (Fig. 1d). On the other hand, Cuffdiff revealed a total of 38,830 genes were identified, in the comparison of F4R negative vs. F4R positive small intestine epithelial cells of Large White piglets, of which 12,810 were novel.

### Differential expression analysis

**Fig. 2:**
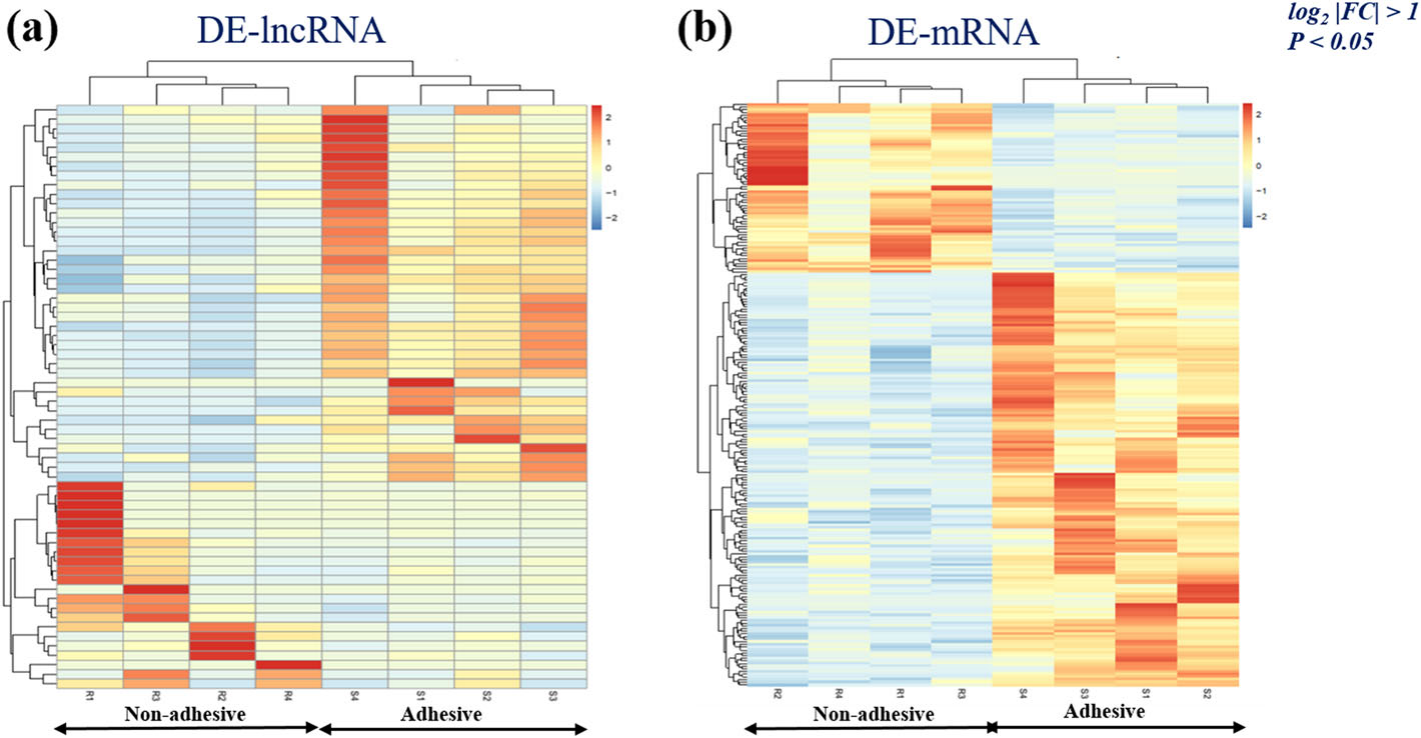
Heatmap of DE-lncRNA and DEGs: **a** Heatmap of differentially expressed long non-coding RNA in the comparison of Non-adhesive Vs. adhesive small intestine epithelial cells. **b** Heat map of differentially expressed genes in comparison of Non-adhesive and adhesive small intestine epithelial cells

To identify differentially expressed lncRNA (DE-lncRNAs) and mRNA (DEGs) from 87,725 transcripts and 38,830 genes detected by cuffdiff respectively, we used log2|FC| > 1and *P* < 0.05 as criteria. Seventy-eight (78) lncRNA transcripts were differentially expressed, 64 up-regulated and 14 down-regulated in the comparison of non-adhesive vs. adhesive porcine small intestine epithelial cells (Fig. 1e, Additional file 3). As for mRNA, 223 were differentially expressed, of which 132 up-regulated and 91 down-regulated (Fig. 1e, Additional file 4). The heatmap of both DE-lncRNA and DE-mRNA showed each group clustered together (Fig. 2a and b) indicating differences in the transcriptional profiles between the two comparisons.

### LncRNA cis- and trans-targets analysis

To predict the coding and non-coding potential of the transcripts, two bioinformatic tools, namely Coding-Non-Coding Index (CNCI) and PLEK.1.2 were used and only transcripts that, were shared in the intersection by two tools were selected. A transcript length ≥ 200 nt, CNCI score < 0 and PLEK score < 0 were used as criteria to evaluate the non-coding potential of lncRNAs. We found 2007 non-coding transcripts shared in the intersection between CNCI and PLEK.1.2 as indicated in the Venn diagram in Fig. 1f. “Seventy (78) lncRNAs were selected from the 2007 lncRNA transcripts, to perform *cis-* and *trans*-target genes analysis using log2 |FC| > 1, *P* < 0.05” (Additional file 3).

The information regarding the strand, sense or antisense of lncRNA transcripts with their exon number and length, and genomic locations are presented in the Additional file 3. The potential *cis* and *trans*-targets of lncRNAs were identified to investigate the potential functions of 78 DE-lncRNAs. For the classification of lncRNA *cis*-target genes, we used the window function in BEDTools.v2.1.2 software to search for *cis*-target genes located within 100 kb upstream and downstream of differentially expressed lncRNAs, and the potentiality of lncRNA as cis-acting was determined. With regard to *cis-*action, 51 DE-lncRNAs corresponded to 194 protein-coding genes (Additional file 5). For the identification of lncRNA *trans-*target genes, we calculated the Spearman correlation between DE-lncRNAs and DE-mRNA using custom scripts (*r* ≥ 0.9 and *P* < 0.05) in R version (3.5.3). Then interactions of DE-lncRNAs and DE-mRNAs with r values ≥0.9 and *P* < 0.05 were selected as *trans*-target genes. With regard to *trans*-action, 7 DE-lncRNAs corresponded to 61 protein-coding genes. Among them, the ETEC-F4ac diarrhea susceptibility-associated genes namely; XLOC_013768 (*KCNMB1*), XLOC_005667 (*GRB2*), and XLOC_025930 (*ACTN4*) (Additional file 6).

### GO and KEGG enrichment of cis- and trans-target genes

**Fig. 3:**
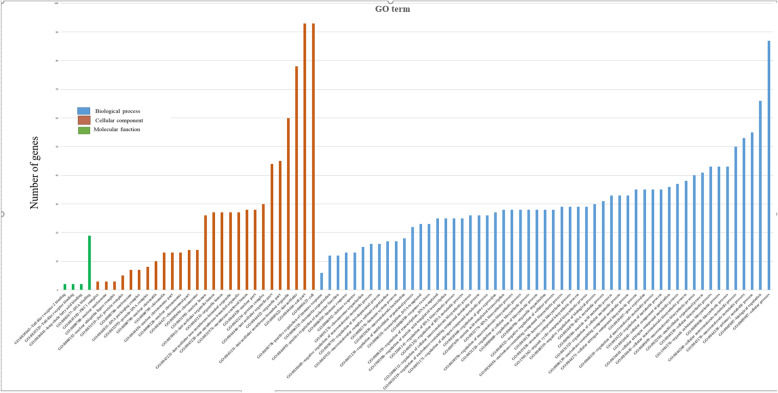
Significantly enriched (*p* > 0.05) GO term: Biological process, cellular component and molecular functions of lncRNA cis-target genes

**Fig. 4:**
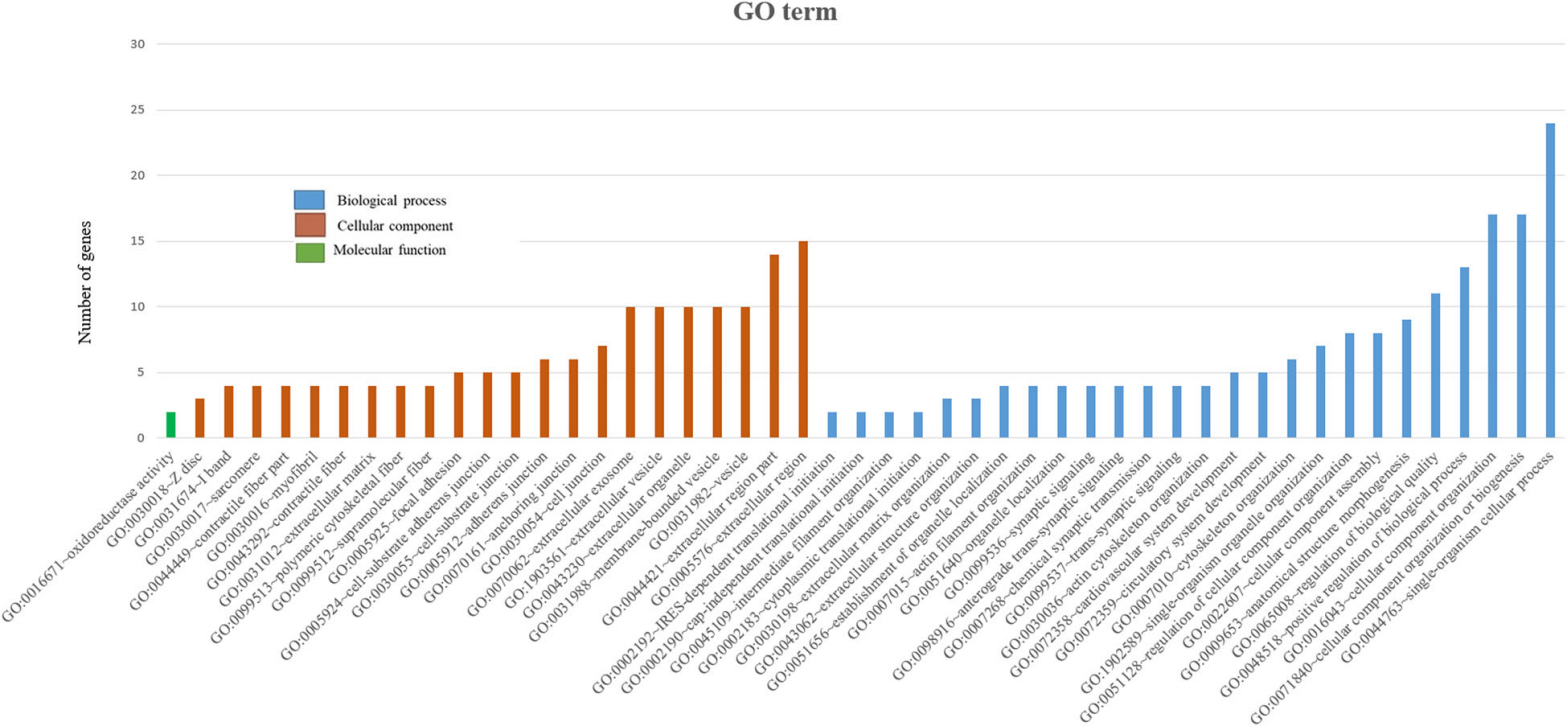
Significantly enriched (*p* > 0.05) GO term: Biological process, cellular component and molecular function of lncRNA trans-target genes

To obtained insight into the biological functions of the 194 *cis*-targets and 86 *trans-*target genes, Gene Ontology (GO) and KEGG pathway analysis were performed using DAVID version 6.8, a web-based software. The terms which are only significantly enriched (*P* < 0.05) GO are listed in Figs. 3 and 4.

The results of GO analysis revealed that *cis-*target DEGs were mostly engaged in cellular process, biological regulation, primary metabolic process, innate immune response, inflammatory response, cytokine production and regulation (Fig. 3 and Additional file 7). In contrast, the *trans*-target genes were mostly involved in single-organism process, cellular component organization, positive regulation of biological process etc. (Fig. 4 and Additional file 8). These results suggest the variation in the transcriptome between the two groups.

KEGG pathway analysis of DE-lncRNAs *cis*- and trans-target genes was performed to predict pathways that are enriched by DE-lncRNA *cis*- and trans-target genes.

KEGG analysis of DE-lncRNAs *cis*-target genes revealed four significantly enriched pathways namely alcoholism, systemic lupus erythematosus, viral carcinogenesis and malaria (Table 1, Additional file 9). Whereas DE-lncRNAs *trans*-target genes were engaged in terms related to ETEC-F4ac adhesion phenotype (among these genes *ACTN4* and *GRB2* were involved in focal adhesion, *KCNMB1* engaged in cGMP-PKG signaling pathway and *ACTN4* was also involved in adherens junction (Table 2 and Additional file 10)) which was detected by the adhesion test in adhesive small intestine epithelial cells (F4R positive pigs). The expression of these three genes were all up-regulated in ETEC-F4R positive (adhesive) piglets compared to ETEC-F4R negative (non-adhesive). This suggests that these genes are candidate genes for adhesion (susceptibility) phenotype to ETEC-F4ac and are regulated via lncRNA *trans*-regulatory mechanism.

**Table 1:** KEGG pathways of lncRNA *cis*-target genes

KEGG pathway	KEGG ID	Genes	*P-*value
Alcoholism	ssc05034	*HIST1H2BN, HIST1H2BC, HIST1H2AJ, HDAC2, ENSSSCG00000023360, ENSSSCG00000024918, ENSSSCG00000020991,, ENSSSCG00000022049*	4.08E-05
Systemic lupus erythematosus	ssc05322	*HIST1H2BN, HIST1H2BC, HIST1H2AJ, ENSSSCG00000023360, ENSSSCG00000024918, ENSSSCG00000020991, ENSSSCG00000022049*	4.65E-05
Viral carcinogenesis	ssc05203	*HIST1H2BN, HIST1H2BC, HDAC2, SLA-1, ENSSSCG00000022049,*	0.0379
Malaria	ssc05144	*HGF, PECAM1, ENSSSCG00000014117*	0.0491

**Table 2:** KEGG pathways of lncRNA *trans*-target genes

KEGG pathway	KEGG ID	Genes
cGMP-PKG signaling pathway	ssc04022	*KCNMB1*
MAPK+D9:CQ9ng pathway	ssc04010	*GRB2*
Focal adhesion	ssc04510	*ACTN4, GRB2*
Adherens junction	ssc04520	*ACTN4*

### Co-expression networks of lncRNAs and their cis and trans-target genes

**Fig. 5:**
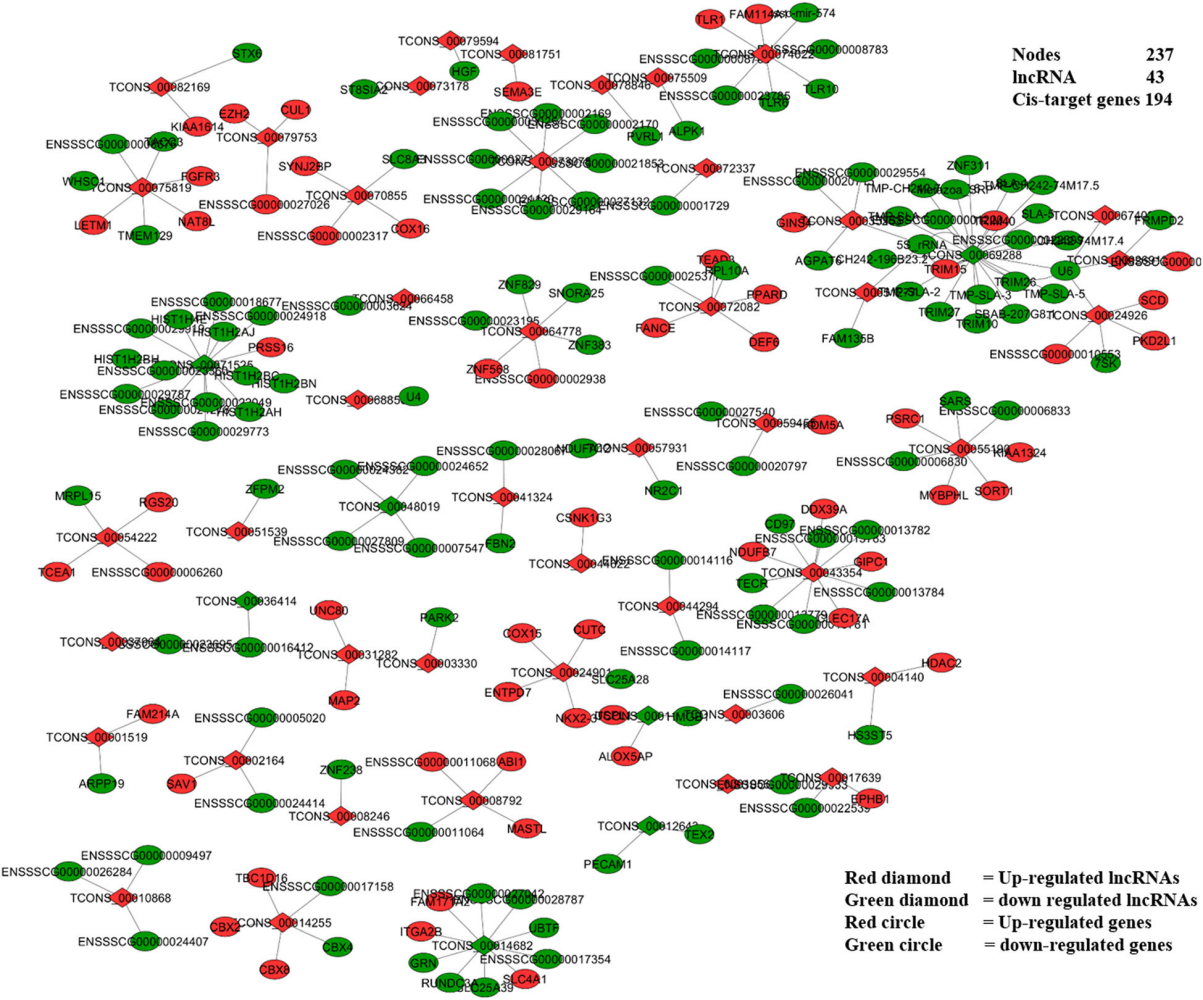
Co-expression network of lncRNAs and their CIS -target genes: LncRNAs and their cis-target genes interaction network comprised 237 nodes. In these networks, red circles are up-regulated genes, green circles are down-regulated genes, red diamonds are up-regulated lncRNAs and green diamonds are down-regulated lncRNAs

**Fig. 6:**
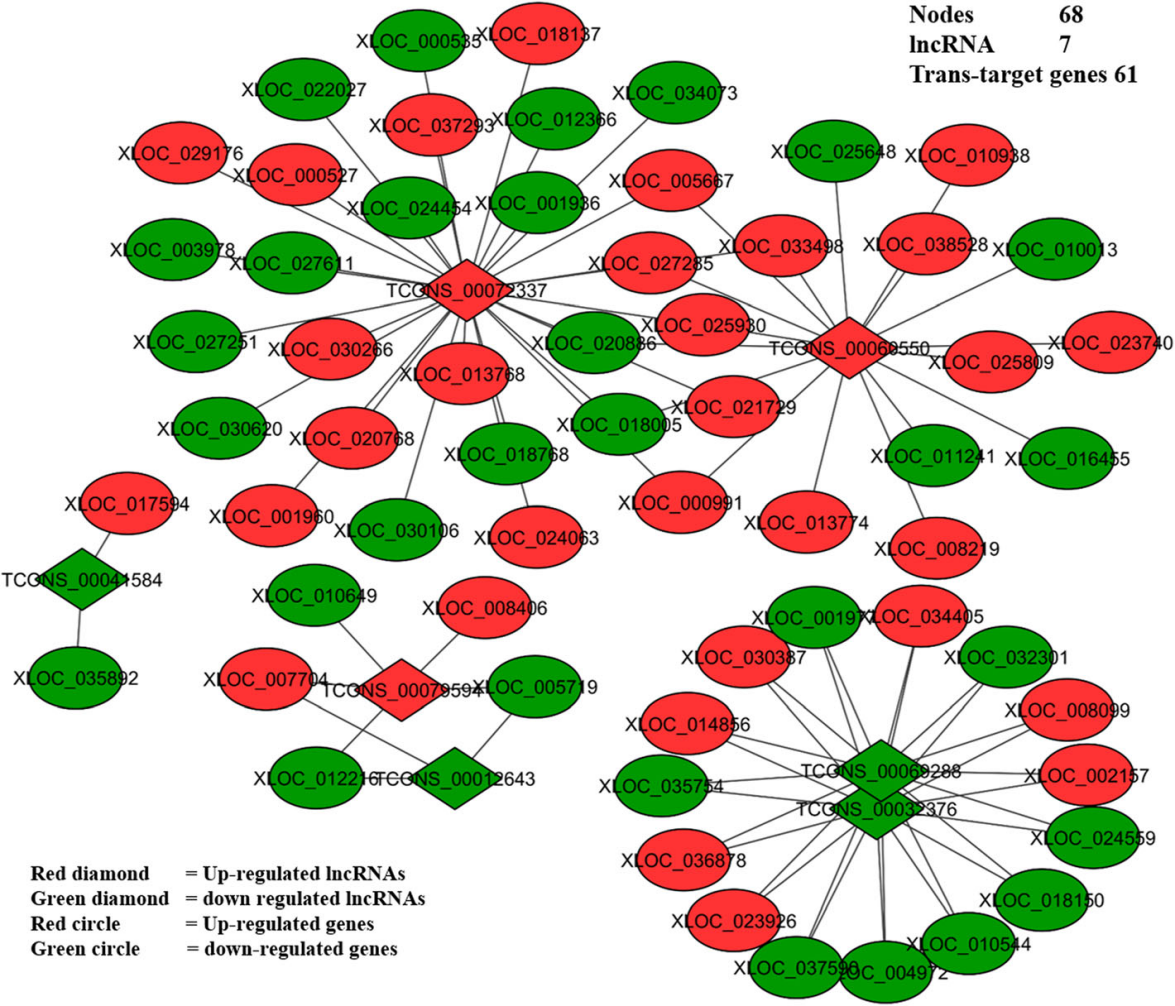
Co-expression network of lncRNAs and their TRNAS-target genes: LncRNAs and their trans-target genes interaction network comprised 68 nodes. In these networks, red circles are up-regulated genes, green circles are down-regulated genes, red diamonds are up-regulated lncRNAs and green diamonds are down-regulated lncRNAs

To elucidate the potential functions of lncRNA, we used DE-lncRNAs and their corresponding differentially expressed *cis*- and *trans-*target genes to construct lncRNA-gene interaction networks using Cytoscape software version (3.5.1). The lncRNAs and their corresponding *cis*-target genes interaction network comprised 237 nodes, including 43 lncRNAs and 194 *cis*-target genes. These nodes formed 191 network pairs (Fig. 5). Whereas the interaction network of lncRNAs and their corresponding *trans*-target genes comprised 68 nodes, including seven lncRNAs (TCONS_00012643, TCONS_00032376, TCONS_00041584, TCONS_00060550, TCONS_00069288, TCONS_00072337, TCONS_00079594) and 61 *trans*-target genes formed 86 network pairs (Fig. 6). These networks show a single lncRNA may be connected to several *cis-* and *trans*-target genes and vice versa. For example, a single lncRNA, LOC102157546 (TCONS_00072337) in component 1 (Fig. 6), is connected to 41 *trans*-target genes including XLOC_013768 (*KCNMB1*). Also TCONS_00060550 in component 2 in the same Fig. 6 is connected to 17 *trans*-target genes including XLOC_005667 (*GRB2*) and XLOC_025930 (*ACTN4*) and a single *trans-*target gene, XLOC_018150 is connected to two lncRNAs (TCONS_00032376 and TCONS_00069288) (Fig. 6), etc.

Therefore the interactions between lncRNAs and their *trans*-target genes likely associate with the development of F4ac adhesion (susceptibility) phenotype detected by the adhesion assay test in F4R positive small intestine epithelial cells. This is because *trans-*target genes such as *KCNMB1* involved in cGMP-PKG signaling pathway, *GRB2* involved in focal adhesion and *ATCN4* engaged in both focal adhesion and adherens junction (Table 2).

### Real-time quantitative PCR (RT-qPCR) validation

To verify the precision and reproducibility of our RNA-seq data, three *trans*-target genes associated with pathways related to F4ac diarrhea susceptibility phenotype, including XLOC_013768 (*KCNMB1*), XLOC_005667 (GRB2) and XLOC_025930 (*ACTN4*) and their corresponding lncRNA regulators, LOC102157546 (TCONS_00072337) targeting *KCNMB1* and TCONS_00060550 (c-Maf inducing protein (CMIP)) regulating both *GRB2* and *ACTN4* were selected for real-time qRT-PCR validation.

**Fig. 7:**
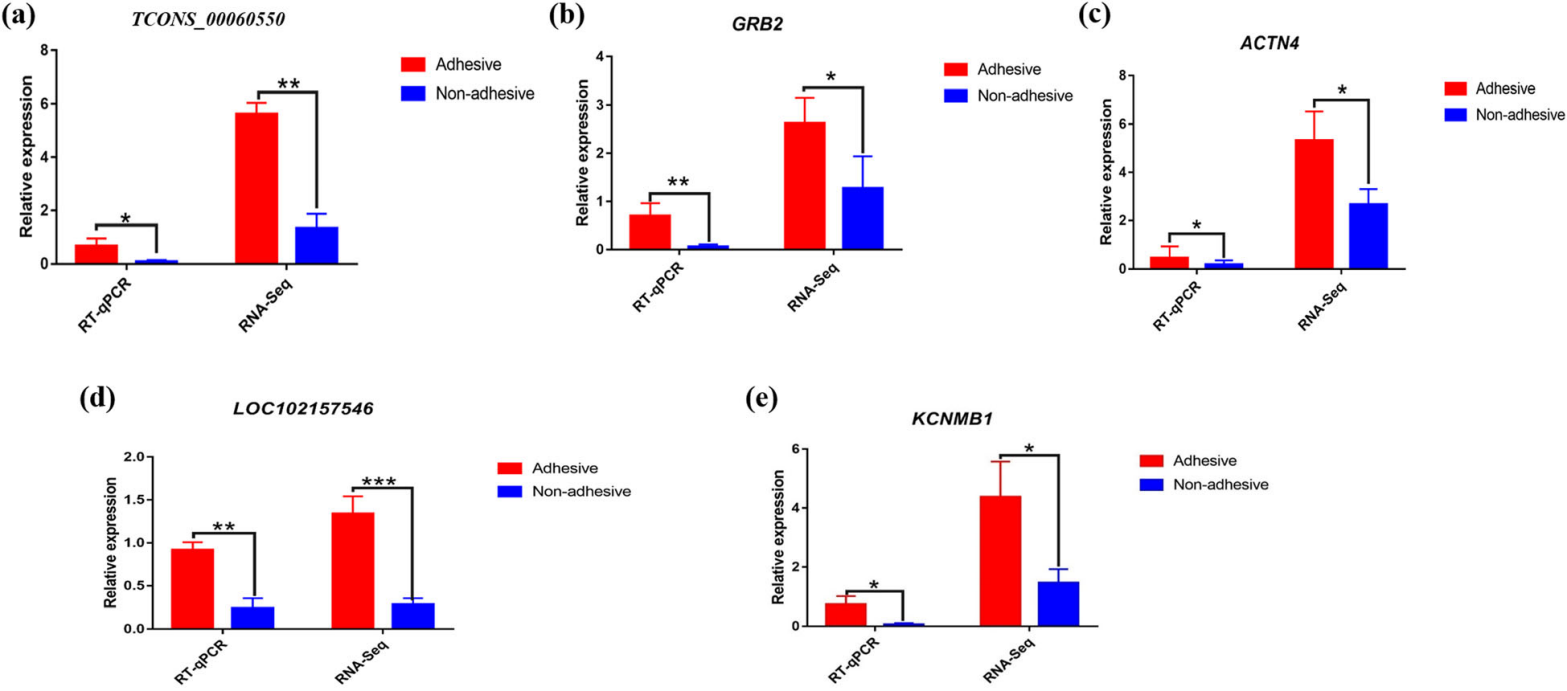
Quantitative real-time PCR validation of two differentially expressed lncRNAs and three corresponding target genes and Venn diagram of lncRNA cis-acting. **a** The expression of TCONS_00060550 (CIMP) was down-regulated in the non-adhesive group compared to the adhesive group. **b** The expression of GRB2 was down-regulated in the non-adhesive group compared to the adhesive group. **c** The expression of ACTN4 was down-regulated in the non-adhesive group compared to the adhesive group. **d** The expression of LOC102157546 was down-regulated in the non-adhesive group as compared to the adhesive group. **e** The expression of KCNMB1 was down-regulated in the non-adhesive group as compared to the adhesive group

The results of RT-qPCR of all two lncRNAs and their three target genes showed similar expression patterns as compared to the RNA-seq data (Fig. 7), suggesting the reliability and the reproducibility of our RNA-seq data.

The positive correlation between lncRNA TCONS_00060550 and its two target genes XLOC_005667 (GRB2) and XLOC_025930 (ACTN4) in Fig. 7a, b and c respectively and the positive correlation between lncRNA TCONS_00072337 (LOC102157546) and its target gene XLOC_013768 (KCNM1) in Fig. 7d and e respectively in the expression patterns, suggest that TCONS_00060550 and TCONS_00072337 (LOC102157546) might play an important role in adhesion of ETEC-F4ac to small intestine epithelial cells which were detected by the adhesion test in F4R positive pigs. None of the target genes and lncRNAs was found negatively correlated in expression regarding association with the phenotype in question.

## Discussion

Over the past few decades, 70–90% of the transcribed mammalian genomes have been identified [11]. Interestingly, roughly, 2% of the mammalian genome are genes encoding proteins, suggesting that non-coding RNA constitutes the majority of the mammalian transcriptome [11], which do not code for proteins but serve regulatory roles [12]. Regarding their length, lncRNAs ranged in size from 200 bp to 100 kb, and have been involved in many biological processes and diseases such as lymphoma in both humans and dogs [13], prostate cancer [14], skeletal muscle development in pig [15] etc. However, the regulatory roles and functions of lncRNAs in the ETEC-F4ac adhesion phenotype in small intestine epithelial cells in piglets remain unclear. To address this matter, in the current study, Illumina high-throughput sequencing was carried out to establish comprehensive lncRNAs and mRNAs profiles of small intestine epithelial cells in Large White piglets differing in their ETEC-F4ac adhesion phenotype and *ITGB5* genotype.

We identified 78 lncRNAs and 223 mRNAs that were differentially expressed in the comparison of F4R negative vs. F4R positive small intestine epithelial cells. This is in consistent with Huang et al. who reported a similar trend in their study on piglet ileum immune response to *C. perfringens* type C infection [4].

LncRNAs function by targeting protein-coding genes. There is growing evidence suggesting that lncRNAs are critical factors in controlling and regulating gene expression via *cis* and *trans*-acting mechanisms [16]. Therefore, to identify lncRNA *cis*-target genes, we employed window function in BEDTools.v2.1.2 software to search for *cis*-target genes located within 100 kb upstream and downstream of differentially expressed lncRNAs. This yielded 51 *cis*-acting lncRNA and 194 corresponding target genes (Additional file 5). Six lncRNAs *cis*-acting are located in both up-stream, down-stream and gene bodies of their target genes (Additional file 5).

Using GO term and KEGG pathway analyses, the *cis*-target genes were found significantly engaged in alcoholism, systemic lupus erythematosus, viral carcinogenesis and malaria. None of the *cis*-target genes was found involved in terms related to ETEC-F4ac diarrhea adhesion (susceptibility) in the comparison of F4R^−^ vs. F4R^+^ Large White piglets. This suggesting that *cis*-acting lncRNAs played not *cis*-regulatory function in controlling their corresponding target genes regarding the adhesion phenotype to ETEC-F4ac which was detected by the adhesion test in F4R positive group.

On the other hand, for *trans*-target genes identification, we used a custom script to calculated Spearman’s correlation coefficient (PCC) between DE-lncRNAs and DE-mRNA. Then lncRNAs-mRNAs interactions with PCC ≥ 0.9 and *P* < 0.05 were selected. This yielded 7 lncRNAs *trans-*acting and 61 corresponding target genes (Additional file 6).

The GO term and KEGG analyses of *trans*-target genes revealed that they were involved in three terms related to adhesion phenotype and diarrhea susceptibility to ETEC-F4ac. These terms were cGMP-PKG signaling pathway, focal adhesion and adherens junction and the trans-target genes were *KCNMB1*, (*ACTN4 and GRB2*) and *ACTN4* respectively (Table 2). The *GRB2* and *ACTN4* are predicted to be the targets of TCONS_00060550 and *KCNMB1* is predicted to be a target of TCONS_00072337 (LOC102157546). This explains that lncRNAs *trans*-acting played trans-regulatory roles in controlling their corresponding target genes regarding the adhesion phenotype and diarrhea susceptibility to ETEC-F4ac which was detected by the adhesion test in F4R positive group.

Potassium calcium-activated channel subfamily M regulatory beta subunit (*KCNMB1*), an ion channel that plays critical functions via its involvement in the homeostasis process of every cell type, has been studied in different organs of different species, the smooth muscle of mice or castrated male swine [17] muscle and lung of human [18], rat and mouse tail and rat saphenous arteries [19], etc. All of these studies and many others have found that the expression levels of *KCNMB1* were greater in infected tissues compared to normal control ones which are coherent with our study.

The up-regulation of *KCNMB1* in F4R+ pig and its involvement in the cGMP-PKG signaling pathway suggesting that it might play a critical role in ETEC-F4ac diarrhea susceptibility which was clearly exhibited by small intestine epithelial cells of piglets in the adhesive group. This is because after consumption of ETEC-F4ac through food or drinking water, the bacteria reach the small intestine of piglets, bind with their F4ac fimbriae to F4R on the surface of intestinal epithelial cells in F4R+ pigs, and establish colonization. Subsequently, after colonization, the bacteria secret enterotoxins called heat-labile (LT) and heat-stable (ST). In cGMP-PKG signaling pathways, the natriuretic peptide receptor 1 (NPR-A and/or NPR-B) at the cell’s surface membrane receives the ST which activates soluble guanylyl cyclase (p-GCs). Then p-GCs catalyzes the cyclization of GTP converting it into cyclic guanosine monophosphate (cGMP). The activation of p-GCs causes increased intercellular cGMP, which in turn, has a direct effect on cGMP-dependent protein kinases (PKG). This interferes with the function of Potassium calcium-activated channel M regulatory beta subunit 1/alfa subunit 1 (*KCNMB1*), that controls and regulates the movements of water and electrolytes in and out of the cell, thus leaving the gate (channel) open allowing more influx of electrolytes and water into the gut lumen causing diarrhea as shown in the mechanism of ETEC-F4ac diarrhea pathogenesis in swine reported in our previous study [10]. Therefore, this elucidating the interactions between host intestinal epithelial cells and virulence factors of enteropathogenic *E.coli* and avail an insight, into how these virulence factors contribute to the adhesion phenotype and diarrhea susceptibility in F4R+ pigs at the molecular level.

Many studies have reported overexpression of growth factor receptor-bound protein 2 (*GRB2*) in many pathological cases in different organs of different species compared to normal controls [20, 21], which is consistent with the current study in which *GRB2* is also overexpressed in adhesive small intestine epithelial cells to ETEC-F4ac.

Similarly, actinin alpha 4 (*ACTN4)* ubiquitous expression in the kidney is also reported in this study to be up-regulated in adhesive small intestine epithelial cells. This is coherent with many studies that reported the overexpression of *ACTN4* in diverse biological conditions including diseases (such as cancers) compared to normal controls [22–24].

Adherens junction is one of the types of adhesion machinery in many cell types in mammals, including epithelial cells. In addition to the regulation of organization within epithelia, adherens junction is also vital in the transmission of information from the external environment to inside the cells [25]. Therefore are necessary for the development and physiology of the epithelial tissues. The involvement of *ACTN4* in adherens junctions pathway reported in this study we strongly believe it might have played a significant role in mediating the attachment of ETEC-F4ac to the small intestine epithelial cells in F4R+ Large White pigs.

On the other hand, focal adhesions are sites of adhesion where cells are connected to the extracellular matrix. It thus mediates the adhesion and enables the communication between them through the physical action of the tight connection [25]. We believe that the mutation at the *ITGB5* (integrin as a cell receptor for F4R) is necessary for the interaction between small intestine epithelial cells and the extracellular matrix environment (including ETEC-F4ac) in adhesion-acquired phenotypes in F4R+ pigs. This is explicitly demonstrated by the involvement of *GRB2* and *ATCN4* in focal adhesion pathway, and which their overexpression is regulated by lncRNA (TCONS_00060550) through trans-regulatory action revealed by this study.

The up-regulation of these three genes in adhesive (F4R+) group might be as a result of the presence of a functional F4R in adhesive small intestine epithelial cells in addition to TT genotype at *ITBG5* SNP that [26] identified in a GWAS study as a promising candidate gene responsible for susceptibility phenotype to both ETEC-F4ab/ac. Therefore, these results provide insights for the phenotypic differences between the two groups with regard to the absence and presence of a functional F4R in the piglets’ small intestine epithelial cells. The differences could also explain the differences in the pigs’ breeding value for adhesion phenotype, the gut microbiome of the animals or other factors that are not explored in this study. We will, therefore, look forward to addressing them in future studies.

In addition, with regard to the limited sample size in this study (four non-adhesive pigs vs. four adhesive pigs), albeit RNA-seq yielded many DE-lncRNA and DEGs in the comparison of F4R negative (non-adhesive) piglets vs. F4R positive (adhesive) piglets, these genes warrant validation in a larger pig population in future studies.

The involvement of *GRB2* in focal adhesion and *ACTN4* in both focal adhesion and adherens junction, these processes are fundamental cellular functions, and are of the utmost importance for the organization of cell structure and shape, leading to normal tissue development and maintenance [27]. Thus, investigation of the molecular mechanisms of cell adhesion may increase our comprehension of the adhesion properties of small intestine epithelial cells which ETEC-F4ac bind to.

With regard to mechanisms by which these genes are regulated, previous studies reported that the up-regulation of *KCNMB1* is regulated by DNA methylation in fibroblast [18] and the up-regulation of *GRB2* is regulated by mir-329 in the pancreas [28] whereas the up-regulation of *ACTN4* is regulated by miR-548b in squamous cells’ carcinoma of the oral tongue in human [29].

Therefore, to decide whether the up-regulation of *KCNMB1, GRB2* and *ACTN4* in adhesive small intestine epithelial cells of Large White piglets reported in the current study regulated by lncRNA via *trans*-regulatory mechanism alone, is sufficient enough to cause the increased expression or combined with other epigenetic regulatory mechanisms? Is yet to be known and therefore warrants further investigation.

To gain insight into how the interactions between lncRNAs and their target genes regulate ETEC-F4ac adhesion phenotype, interaction networks comprised of DE-lncRNAs and their predicted *cis* and *trans-*acting targets genes were constructed. These networks show a single lncRNA may be connected to several *cis*- and *trans*-target genes and vice versa [30]. Based on the networks, XLOC_013768 (*KCNMB1*) is predicted to be a target of TCONS_00072337 (LOC102157546), and XLOC_005667 (*GRB2*) and XLOC_025930 (*ACTN4*) are predicted to be targets of TCONS_00060550 (Fig. 6). This implies that these two lncRNAs might play an important role in the expression of the adhesion phenotype detected by the adhesion test in Large White F4R positive pigs.

## Conclusion

This study is the first to disclose the association of these lncRNAs and their target genes with the adhesion of ETEC-F4ac to small intestine epithelial cells of Large White piglets.

In addition to the insights into the biology of the phenotypes of ETCE-F4ac adhesion to the small intestine epithelial cells of F4R+ pigs, the results also provide insights into epigenetic regulatory roles on key genes associated with adhesion phenotype and susceptibility of small intestine epithelial cells of Large White piglets to ETEC-F4ac induced diarrhea. Therefore, this study provides a foundation for future studies to build on these findings and to verify the regulatory effects of lncRNAs on their *cis*- and *trans*-target genes. In general, the results provide an important theoretical basis and experimental data which could be used in a breeding program to develop F4R negative pigs in the swine world.

## Methods

### Ethics statement

All the experiments carried out in this study followed the Animal Welfare Committee’s approved protocol of Agricultural University (Permit Number: DK996). Some of the methods described here-under in this study were carried out in our previously published paper [10].

### Samples collection

The Large White piglets used in this study were raised in the experimental farm at Chinese Academy of Agricultural Sciences. This breed is a domestic pig breed originated in Yorkshire in the UK and is now the most reared commercial pig worldwide. All the piglets used in this study were healthy individuals, i.e. no diarrhea infection was noticed. The piglets were slaughtered exactly at the weaning age of 35 days. Piglets were first humanely euthanized by using Carbon dioxide (CO2) inhalation method prior to slaughter. Briefly, the piglets needed to be euthanized were identified, and then rectal temperature, weight, sex and identification of each piglet were recorded. To make the piglets unconscious in the shortest possible time, the gas chamber (61 cm × 38 cm × 46 cm) with a sealable lid and a gas inlet was pre-filled with CO2, then the piglets in groups of four each were placed into the chamber. Gas was continuously pumped into the chamber, and the piglets became unconscious. To confirm this unconsciousness, palpebral reflex and response to pinprick on the nose were performed every 30 s after the piglet assumed a loss of posture. The piglets were confirmed dead after there were no more palpebral reflexes and breathing. The animals were then slaughtered and the samples of small intestine tissue from each animal were aseptically collected within 30 min after slaughter. Each tissue sample was cut longitudinally, rinsed with EDTA (5 mmol/ L EDTA, pH 7.4) a hypotonic solution, placed in a liquid nitrogen container, then immediately transferred to the laboratory, put in the freezer at − 80^0^ C upon arrival and kept until used for RNA extraction and adhesion assay later. For more detail on sampling, animal population and slaughtering procedures among others can be found in our previous study [1].

### Adhesion test

Adhesion herein is the tendency of ETEC-F4 fimbriae to bind or attach to intestinal epithelial cells F4 receptors in adhesive (susceptible) piglets. The adhesion (susceptibility) phenotype of 161 Large White piglets’ small intestine epithelial cells to ETEC-F4ac strain 200 (C83907, O149:K91) was carried out using in vitro adhesion test. This strain was obtained from the China Institute of Veterinary Drug Control, Beijing, China. The adhesion assay was done by the research group in our lab, therefore, for more details on the procedures of the in vitro adhesion test and the results of the adhesion assay can also be found in our previous study [1]. Briefly, the brush border cell suspension and the bacterial suspension (0.1 mL each) were mixed with 0.4 mg/mL mannose and incubated for 30 min at room temperature. Subsequently, phase-contrast microscopy was used for checking the adhesion in a drop of the suspension mixture. A single epithelial cell was deemed adhesive when more than five bacteria were adhering to the brush border membrane. From the epithelial cell specimen of each sample, more than twenty (20) epithelial cells were checked and the piglet was deemed as strongly adhesive when > 80% of the epithelial cells were adhesive. Adhesive when 10–80% of the epithelial cells were adhesive. When > 10% of the cells were adhesive, they were considered as weakly adhesive, and when no epithelial cells were adhesive, they were regarded as non-adhesive.

The terms adhesive and non-adhesive will be used interchangeably with susceptible and resistant respectively, in the text. Because it is widely believed that susceptibility and resistance of small intestine epithelial cells to diarrhea induced by ETEC-F4 is based on the presence and absence of F4R respectively.

### Genotyping of piglets

After adhesion assay, to confirm the genetic architecture (genotype-phenotype), the genotypes of the piglets with F4R and without F4R on their small intestine epithelial cells brush border, was sequenced by our previous study [31] using integrin subunit beta 5 (*ITGB5*) SNP NC_010455.5 (g.135577826 C > T) which was identified as an important candidate gene of F4R in a GWAS study by Fu et al. 2012. Recently this gene also has been reported by Wang et al. (2019) as playing a key role in the ETEC-F4ac susceptibility phenotype in piglets diarrhea [32].

### Experimental animals

The animals used in this study were selected from 161 animals used by our previous study [1] to perform adhesion assay. These animals were selected based on three criteria namely; adhesion phenotypes (adhesive and non-adhesive), *ITGB5* SNP (C > T) genotypes and sex of the animals. The above criteria yielded eight (8) male piglets, four (4) pigs with adhesive small intestine epithelial cells with TT genotype were designated as the adhesive group and another four (4) pigs with non-adhesive small intestine epithelial cells with CC genotype were designated as the non-adhesive group.

### Total RNA extraction and quality examination

Briefly, total RNA was isolated from eight small intestine epithelial cells samples, four from each group (non-adhesive and adhesive) using Trizol Reagent (Invitrogen, Carlsbad, CA, USA) following manufacturer’s protocol. The concentrations of the total RNA were determined using the NanoDrop spectrophotometer. The checking of RNA purity was performed using the kaiaoK5500®Spectrophotometer (Kaiao, Beijing, China). The contamination and degradation of RNA quality were checked on 1% agarose gels revealed three distinct bands of 28S, 18S and 5S (Additional file 1, Figure S1). The concentration and integrity of all the RNA samples were good enough (OD260/280 > 1.90 and RNA integrity number > 8.7) for doing the sequencing. Then 20 μL from the isolated total RNA from all the samples were sent to the Annoroad Gene Technology Corporation company in Beijing for sequencing. The rest were stored in the freezer at − 80 °C until used for cDNA synthesis to validate some genes using RT-qPCR.

### Library preparation for RNA sequencing

A total amount of two μg RNA per sample was used as input material for the RNA sample preparations. Sequencing libraries were generated using RNA library preparation kit for Illumina following the manufacturer’s recommendations. Briefly, the purification of mRNA from total RNA was performed using poly-T oligo-attached magnetic beads. More details about library preparation can be found in our previously published paper [10]. The libraries were sequenced and 150 bp paired-end reads were generated using an Illumina platform (HiSeq Xten).

### RNA-Seq data analysis

The raw dataset used here in this study was previously used to carry out a differential expression analysis in our previous published paper [10]. Briefly, the clean reads (clean data) were obtained by removing reads containing more than 5% Ns, reads containing adapters, low quality reads reads and with Qphred < 30 from raw reads. RNA-Seq paired-end clean data obtained were then analyzed using Hisat2–2.1.0., Samtools-1.9, Stringtie 1.3.5 and cufflinks-2.2.1. Porcine reference genome (*Sus scrofa* 11.1) was obtained from Ensembl (ftp://ftp.ensembl.org/pub/release-96/fasta/sus_scrofa/dna/).

Then reference genome index was created by the build-index function in Hisat2–2.1.0 software (http://ccb.jhu.edu/software/hisat2) package with default options. The alignment of paired-end reads from each sample to the reference genome was performed using Hisat2–2.10 with default settings.

After the alignment, the generated SAM files were sorted to BAM files using Samtools 1.9 (http://samtools.sourceforge.net). Subsequently, Stringtie 1.3.5 was used to assemble the transcripts using BAM files as inputs. Then the generated transcripts were merged using cuffmerge, followed by cuffcompare. After this, the transcripts with length ≥ 200 nt were filtered and used as inputs in CNCI and PLEK1.2 for coding and non-coding prediction. The CNCI score < 0 and PLEK1.2 < 0 were used as criteria for selecting non-coding transcripts/isoforms. The selected non-coding transcripts/isoforms from the two software tools were used as input to draw the Venn diagram and those transcripts/isoforms which were in the intersection between the two software tools were selected for *Cis* and *Trans* target genes prediction. The results of Cuffcompare were also used for screening the position of lncRNA transcripts/isoforms based on their class codes including intronic antisense, intronic sense, exonic anti-sense, and exonic sense, etc. Then Cuffdiff function, the differential expression analysis tool in Cufflinks-2.2.1 software, was employed to detect the expression levels of the isoforms/transcripts and genes as FPKM (fragments per kilobase of transcript per million mapped reads). Differentially expressed lncRNAs and mRNAs in comparison of non-adhesive vs. adhesive groups were identified using *P* < 0.05 and log2 |FC| > 1 as the cut-off points. Raw sequencing data analyzed in this study have been uploaded to the Sequence Read Archive (SRA) in NCBI and can be found under accession number: PRJNA562774 or (Https://www.ncbi.nlm.nih.gov/Traces/study/?acc=PRJNA562774).

### LncRNA cis- and trans-target genes prediction analyses

The basic principle of predicting if a lncRNA targets a gene is that, lncRNAs are regulators of protein-coding genes that lie near their genomic locations or coordinates. Therefore, the protein-coding genes near the location of lncRNA transcripts/isoforms (upstream and downstream 100 k) were screened out as its potential *cis*-regulatory targets. Differentially expressed lncRNAs (log2 |FC| > 1, *P* < 0.05) were selected for *cis*- and *trans*-target gene prediction, and then were integrated with differentially expressed mRNA data for the improvement of precision of target prediction. For classifying lncRNA *cis*-target genes, we used the window function in BEDTools.v2.1.2 software to search for *cis*-target genes located within 100 kb upstream and downstream of differentially expressed lncRNAs, and the potentiality of the lncRNA as *cis*-acting was determined. To identify lncRNA as *trans*-target genes, we calculated the Spearman correlation coefficient (PCC) between DE-lncRNAs and DE-mRNA using custom scripts (*r* ≥ 0.9 and *P* < 0.05).

### LncRNA-gene interaction networks

The differentially expressed lncRNAs and their corresponding *cis*- and *trans*-target genes were used to construct a regulatory network using a standard tool for integrated analysis and visualization of biological networks called Cytoscape (v-3.5.1).

### Gene ontology (GO) annotation and KEGG analysis

Biological process enrichment analyses for the predicted *cis*- and *trans*-target genes of the differentially expressed lncRNAs were performed using a web-based software called Database for Annotation, Visualization and Integrated Discovery (DAVID) version 6.8 (Https://david.ncifcrf.gov/content.jsp?file=DAVID_Publications.h,). We used the Ensembl IDs of all *Cis* and *Trans* target genes and uploaded them separately in DAVID software, then we selected ENSEMBL_GENE_ID from the list of identifiers in DAVID software. Subsequently, we selected the gene list from the list type and submitted it. Species *Sus scrofa* was used as background.

### Complementary DNA synthesis and quantitative real-time -PCR (RT-qPCR)

The synthesis of cDNA from total RNA was performed using PrimeScriptTM RT reagent kit following the manufacturer’s instructions as described in our previously published paper [10]. Reverse transcription reactions were carried out in a final volume of twenty (20) μL. The PCR reaction conditions were 37 °C for 15 min and 85 °C for 5 s and then stored at − 20 °C for RT-qPCR later.

Reactions of real-time quantitative PCR were performed in a final volume of twenty (20) μL using Roche SYBR Green PCR Kit with a Light-CyclerH 480 Real-Time PCR System (Roche, Hercules, CA, USA). The porcine GAPDH was used as the internal standard to adjust the input of cDNA and to normalize the expression.

RT-qPCRs were conducted on each cDNA (duplicate) sample and the average Ct was used for the analysis. Briefly, the ΔCt was obtained by finding the difference between the reference housekeeping gene and the test gene. The ΔΔCt was calculated by finding the difference between samples. The relative expression levels of genes were analyzed against the swine GAPDH gene using the 2 − △△Ct method. The primers used to amplify two lncRNAs and three *trans*-target genes were designed using Primer3 online software (https://www.ncbi.nlm.nih.gov/tools/primer-blast/index.cgi?) and were further checked for primer-dimer and primer self-complementarity using oligo6 software Version 6.41. The primers used for validation are listed in Table 3. The melting curve with a single peak was used to validate the specificity of the RT-qPCR amplification. GraphPad Prism version 7.00 was used for calculating the statistical differences and draw graphs.

**Table 3:** Primers for RT-qPCR validation of trans-target genes and their regulatory lncRNA in small intestine epithelial cells in Large White piglets

lncRNA	Primer (5′ - 3′)	Size	Target gene	Primer (5′-3′)	Size
(TCONS_00060550) (CMIP)	F CCTGCCTTCCCTGTGTGG R GCTCCCCTCCAGTCCCATA	125	*GRB2*	F ATCCGTCTCCAGAAACCAGCA R GGGTCAAAGTCAAAGA	93
			*ACTN4*	F CAGCTGCTCACCACCATCG R ACTCCTGCATCTGCTCTTGG	97
TCONS_00072337 (LOC102157546)	F CAGCAGGAGTGGAAGATGGT R TGACCTTGGCACAGCGAATA	186	*KCNMB1*	F TACTACATCCTGGGCACGAC R CTGGTCCCTGATGTTGGTCTC	99

## Supplementary Information

**Additional file 1: Figure S1** of RNA quality.

**Additional file 2: Table S1** Summary of RNA sequencing.

**Additional file 3.** Total number of identified isoforms and DE-lncRNA.

**Additional file 4.** Total number of identified genes and DEGs.

**Additional file 5.** DE-lncRNAs and their cis-target genes.

**Additional file 6.** DE-lncRNA and their trans-target genes.

**Additional file 7.** GO terms, biological processes, cellular components and molecular function of cis-target genes.

**Additional file 8.** GO terms, biological processes, cellular components and molecular function of trans-target genes.

**Additional file 9.** KEGG of cis-target genes.

**Additional file 10.** KEGG of trans-target genes.

## Data Availability

Raw sequencing data for the eight samples analyzed in this study have been uploaded to the Sequence Read Archive (SRA) in National Center for Biotechnology Information (NCBI), and are available under accession number: PRJNA562774 or (Https://www.ncbi.nlm.nih.gov/Traces/study/?acc=PRJNA562774). Data generated during analysis are included in the manuscript as supplementary files.

## Abbreviations

ETEC: Eterotoxigenic *Escherichia coli*
F4ac: Fimbriae 4 ac antigen varian
F4ab: Fimbriae 4ab antigenic varian
lncRNAs: Long non-coding RNAs
F4R: Fimbriae 4 receptors
RNA-seq: RNA sequencing
*ITGB5*: Integrin subunit beta 5
DEGs: Diferentially expressed genes
*KCNMB1*: Potassium calcium-activated channel subfamily M regulatory beta subunit
*GRB2*: Growth factor receptor bound protein 2
*ACTN4*: Actinin alpha 4
GWAS: Genmome Wide Association Study
mRNAs: Messenger RNAs
SNP: Single Nucleotide Polymorphism
ST: Heat Stable
*E. coli*: *Escherichia coli*
cDNA: Complementary DNA
EDTA: Ethylenediaminetetraacetic acid
pH: Hydrogen ion concentration
RT-qPCR: Real-time quantitative PCR
dNTPs: Deoxyribose nucleoside triphosphate
PCR: Polymerase chain reaction
NCBI: National Center for Biotechnology Information
SRA: Sequence Read Archive
GO: Gene ontology
KEGG: Kyoto Encyclopedia of Genes and Genomes
DAVID: Database for Annotation, Visualization and Integrated Discovery
GAPDH: Glyceraldehyde-3-Phosphate Dehydrogenase
rRNA: Ribosomal RNA
FPKM: Fragments per kilobase of transcript per million mapped reads
cAMP: Cyclic adenosine monophosphate
cGMP-PKG: Cyclic guanosine monophosphate-protein kinase G
CNCI: Coding-Non-Coding Index
PCC: Pearson’s correlation coeffient
