# Supplementary material for: Integrated analysis of lncRNAs and mRNAs reveals key trans-target genes associated with ETEC-F4ac adhesion phenotype in porcine small intestine epithelial cells
Source: BMC Genomics. 2020 Nov 10;21:780. doi: 10.1186/s12864-020-07192-8 (PMC7653856; doi:10.1186/s12864-020-07192-8)
Supplement: Supplementary file 1 — Additional file 1: Figure S1 of RNA quality. [file 12864_2020_7192_MOESM1_ESM.pdf]

Figure S1: The quality of RNA

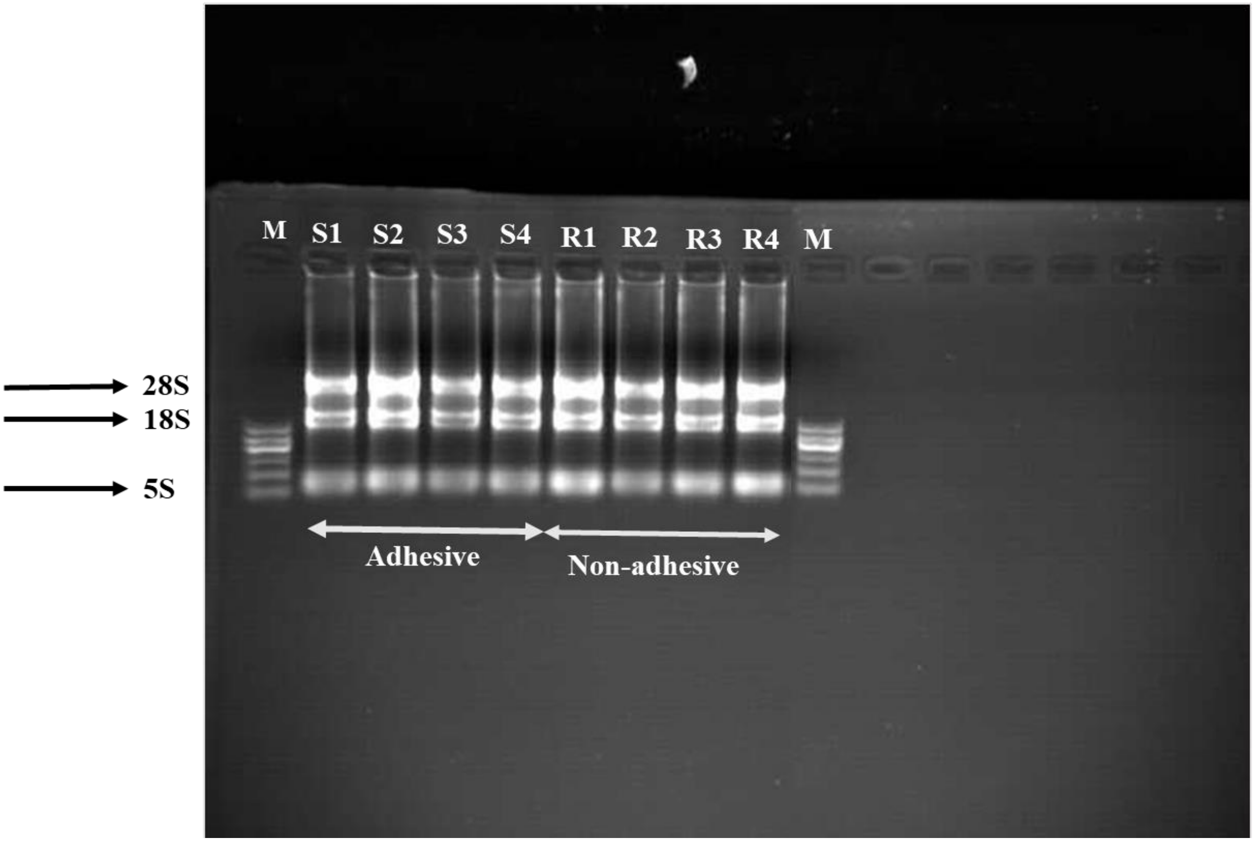

Figure S1. The RNA quality on 1% agarose gels showing three distinct bands of 28S, 18S and 5S

M: Marker

S1 – S4 : Susceptible individuals

R1 – R4: Resistant individuals
