# Supplementary material for: Integrated analysis of lncRNAs and mRNAs reveals key trans-target genes associated with ETEC-F4ac adhesion phenotype in porcine small intestine epithelial cells
Source: BMC Genomics. 2020 Nov 10;21:780. doi: 10.1186/s12864-020-07192-8 (PMC7653856; doi:10.1186/s12864-020-07192-8)
Supplement: Supplementary file 2 — Additional file 2: Table S1 Summary of RNA sequencing. [file 12864_2020_7192_MOESM2_ESM.pdf]

Table S1. Total number of raw reads, clean reads and percentage of clean reads, alignment rate in each sample

	Non-adhesive group				Adhesive group			
Sample	R1	R2	R3	R4	S1	S2	S3	S4
Raw Reads	123574890	115189226	127370354	122342616	126569978	130924082	126760832	126165252
Clean Reads	121798254	113294846	125627966	120851962	124768650	129340552	124748322	124240216
Clean Reads (%)	98.56	98.36	98.63	98.78	98.58	98.79	98.41	98.47
Alignment rate (%)	95.70	96.17	97.14	96.12	97.28	96.54	97.16	96.57
